# Evaluation of an Anti-Tumor Necrosis Factor Therapeutic in a Mouse Model of Niemann-Pick C Liver Disease

**DOI:** 10.1371/journal.pone.0012941

**Published:** 2010-09-23

**Authors:** Melanie Vincent, Naomi L. Sayre, Mark J. Graham, Rosanne M. Crooke, David J. Shealy, Laura Liscum

**Affiliations:** 1 Department of Physiology, Tufts University School of Medicine, Boston, Massachusetts, United States of America; 2 Cardiovascular Disease Antisense Drug Discovery, Isis Pharmaceuticals, Inc, Carlsbad, California, United States of America; 3 Centocor Research and Development, Inc., Radnor, Pennsylvania, United States of America; National Institutes of Health, United States of America

## Abstract

**Background:**

Niemann-Pick type C (NPC) disease is a lysosomal storage disease characterized by the accumulation of cholesterol and glycosphingolipids. The majority of NPC patients die in their teen years due to progressive neurodegeneration; however, half of NPC patients also suffer from cholestasis, prolonged jaundice, and hepatosplenomegaly. We previously showed that a key mediator of NPC liver disease is tumor necrosis factor (TNF) α, which is involved in both proinflammatory and apoptotic signaling cascades. In this study, we tested the hypothesis that blocking TNF action with an anti-TNF monoclonal antibody (CNTO5048) will slow the progression of NPC liver disease.

**Methodology/Principal Findings:**

Treatment of wild-type C57BL/6 mice with NPC1-specific antisense oligonucleotides led to knockdown of NPC1 protein expression in the liver. This caused classical symptoms of NPC liver disease, including hepatic cholesterol accumulation, hepatomegaly, elevated serum liver enzymes, and lipid laden macrophage accumulation. In addition, there was a significant increase in the number of apoptotic cells and a proliferation of stellate cells. Concurrent treatment of NPC1 knockdown mice with anti-TNF had no effect on the primary lipid storage or accumulation of lipid-laden macrophages. However, anti-TNF treatment slightly blunted the increase in hepatic apoptosis and stellate cell activation that was seen with NPC1 knockdown.

**Conclusions/Significance:**

Current therapeutic options for NPC disease are limited. Our results provide proof of principle that pharmacologically blocking the TNF-α inflammatory cascade can slightly reduce certain markers of NPC disease. Small molecule inhibitors of TNF that penetrate tissues and cross the blood-brain barrier may prove even more beneficial.

## Introduction

Niemann-Pick type C (NPC) disease is an autosomal recessive lysosomal storage disease characterized by the accumulation of cholesterol and glycosphingolipids. Ninety-five percent of clinical cases are caused by mutations in the *NPC1* gene [1]. Symptoms of NPC include vertical gaze palsy, ataxia, dystonia and progressive neurodegeneration [2]. The majority of NPC patients die in their teen years due to their neurodegeneration; however, their liver disease is also significant [3]. Approximately half of NPC patients suffer from cholestasis, prolonged jaundice and hepatosplenomegaly [1], [4], [5]. NPC1-deficient mice show hepatic cholesterol accumulation, hepatomegaly, elevated serum liver enzymes, and increased apoptosis [6], [7], [8], [9], [10], [11]. Lipid-laden macrophages accumulate and recruit inflammatory cells [11]. Stellate cells proliferate and deposit collagen, leading to fibrosis. The mechanism by which NPC1 dysfunction leads to liver disease is unknown.

We previously showed that tumor necrosis factor (TNF) α is a key mediator of NPC liver disease [12]. TNF-α is an inflammatory cytokine that is secreted by foamy macrophages. It recruits inflammatory cells, stimulates hepatic stellate cells, and activates an apoptotic signaling cascade. We determined that liver-specific knockdown of NPC1 in TNF-α deficient mice leads to attenuated hepatocyte apoptosis, fibrosis and foamy macrophage clustering into granulomas.

In this study, we have tested the hypothesis that blocking TNF-α action with an anti-TNF-α monoclonal antibody (CNTO5048) will slow the progression of NPC liver disease. Targeting TNF-α mediated inflammation is not expected to halt the primary lysosomal lipid accumulation, but it may reduce secondary consequences of NPC liver disease. Our results indicate that anti-TNF treatment has only a modest effect in blunting the hepatic apoptosis and stellate cell activation that is characteristic of NPC disease.

## Results

### Anti-TNF suppresses the TNF-α response induced by an LPS challenge

To ensure that anti-TNF is efficacious in our model system, we treated wild-type mice for 7 days with either anti-TNF or saline. We then challenged the mice with injection of either lipopolysaccharide (LPS) or saline. The pathological effects of LPS are, in part, mediated by the release of TNF-α [13]. We assessed the ability of the monoclonal antibody to neutralize TNF-α by measuring the plasma concentration of the downstream pro-inflammatory cytokine, IL-6, three hours after LPS injection.

Control mice treated with saline injections and no LPS challenge had undetectable plasma levels of IL-6 (<4 pg/ml). Two mice treated with saline injections and then subjected to an LPS challenge had 68 and 74 ng/ml of plasma IL-6. Two mice treated with anti-TNF injections and then subjected to an LPS challenge had reduced levels of plasma IL-6, at 27 and 29 ng/ml.

Hepatic NPC1 knockdown in mice caused TNF-α-mediated hepatic inflammation that was markedly less severe than that seen with LPS treatment. We injected three mice with NPC1-specific antisense oligonucleotides (ASOs) twice a week for 15 weeks to induce NPC liver disease [14] and found undetectable levels of IL-6 (<4 pg/ml). Since anti-TNF was able to blunt the massive inflammatory response elicited by LPS, we reasoned that it would be able to suppress the more modest inflammation that results from hepatic NPC1 knockdown.

### Anti-TNF treatment of NPC1 knockdown mice does not alleviate hepatic lipid storage

**Figure 1:**
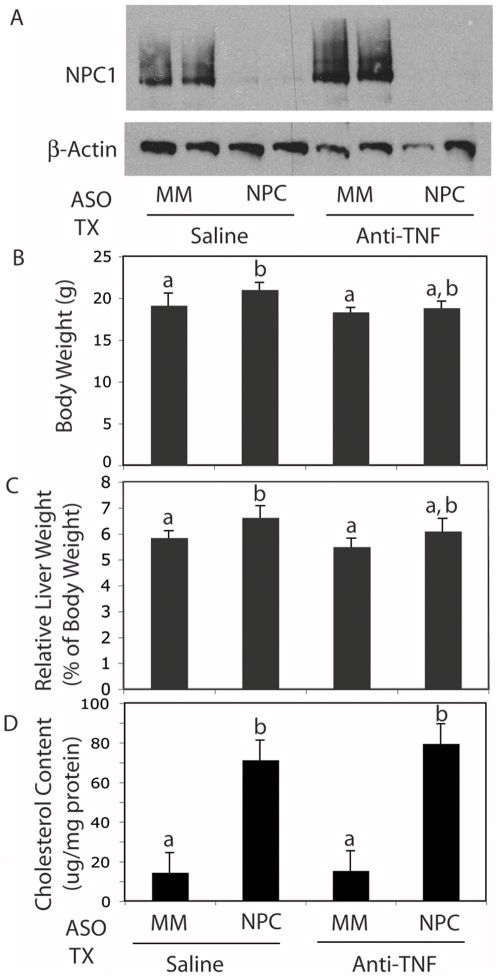
Effect of NPC1 knockdown and anti-TNF treatment on NPC1 protein levels, whole body weight, liver weight, and hepatic cholesterol content. A: Expression of NPC1 in livers of mice injected for 9 weeks with mismatched (MM) and NPC1-specific ASOs and subjected to treatment (TX) with saline or anti-TNF. β-Actin was used as a loading control. Whole body weight (B), relative liver weight (C) and hepatic unesterified cholesterol content (D) in mice injected with mismatched (MM) and NPC1-specific ASOs and subjected to treatment (TX) with saline or anti-TNF. Each bar represents the mean ± SD of 5 animals in each treatment group. The lettering (a, b) shows statistically dissimilar groups. Samples with two letters represent values that are intermediate to the statistical groups and are thus not considered significantly different from either group.

Our experimental protocol had twenty C57BL/6 mice divided into four treatment groups. Ten mice each were injected twice a week with NPC1-specific ASOs or with mismatched control ASOs. Five mice in each group were injected once a week with saline or with the anti-TNF-α monoclonal antibody. NPC1 protein levels in the liver were knocked down to less than 10% of control levels by NPC1 ASO treatment (Fig. 1A). Anti-TNF treatment had no effect on NPC1 protein levels (data not shown). NPC1 knockdown in saline-treated mice led to a small but significant increase in body and liver weights (p<0.05) when compared to mismatched ASO controls (Fig. 1B and 1C). NPC1 knockdown in anti-TNF-treated mice led to a small increase in body and liver weights.

Previously, we showed that TNF-α does not play a role in the development of the primary storage phenotype of NPC [12]. Our new results are consistent with the previous report. Here we found that NPC1 knockdown led to a five-fold increase in hepatic unesterified cholesterol content (p<0.01) (Fig. 1D). The same increase in hepatic cholesterol content was seen when NPC1 was knocked down in anti-TNF-treated mice. Thus, as expected, the anti-TNF treatment had no effect on the cholesterol storage that results from NPC1 dysfunction.

Serum alanine aminotransferase (ALT) activity was measured in the four treatment groups to determine whether anti-TNF treatment of NPC1 knockdown mice resulted in less liver injury. Mice injected with mismatched ASOs and treated with saline or anti-TNF had similar serum ALT activities of 23.6±4.5 and 25.2±5.4 U/L, respectively. NPC1 knockdown led to a significant increase in serum ALT to 98±12.9 U/L. The serum ALT activity of NPC1 knockdown mice was not significantly reduced by anti-TNF treatment (78.3±20.7 U/L). These data indicate that anti-TNF has no effect on the hepatic injury that leads to enzyme release.

**Figure 2:**
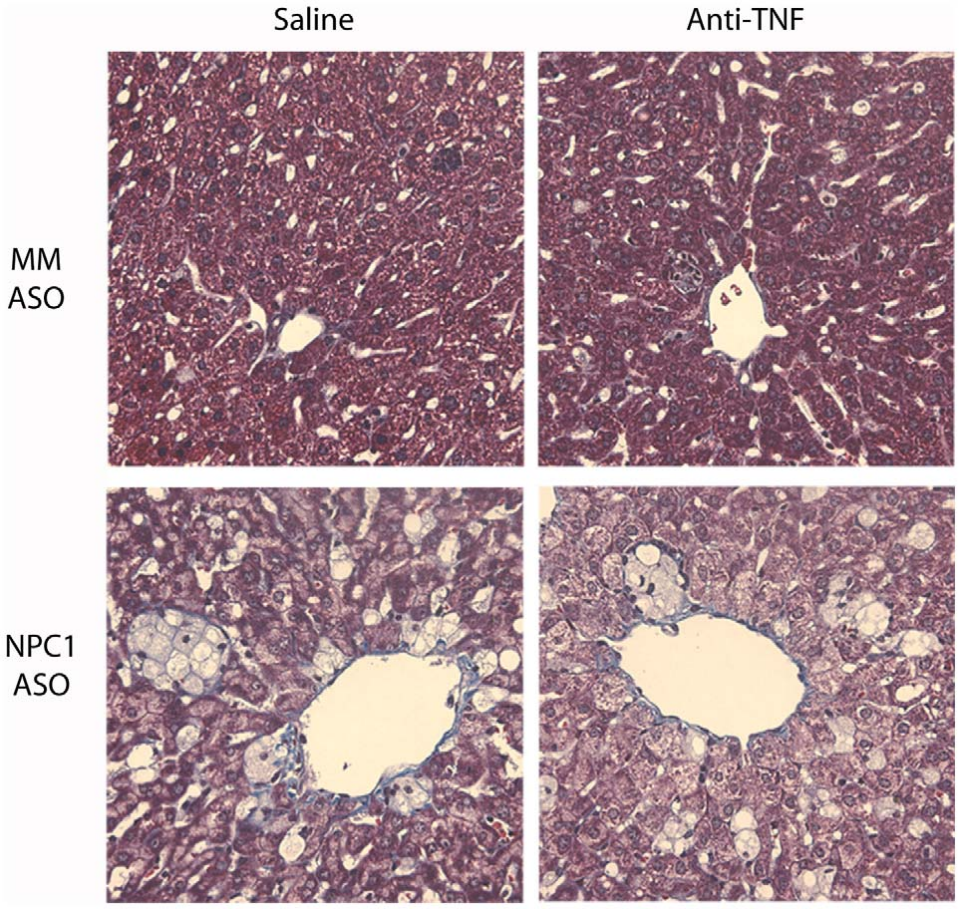
Hepatic formation and clustering of foamy macrophages in NPC1 knockdown mice. Masson's trichrome stained liver sections from mice injected for 9 weeks with mismatched (MM) and NPC1-specific ASOs and subjected to treatment with saline or anti-TNF. Images were photographed at 200X magnification.

**Figure 3:**
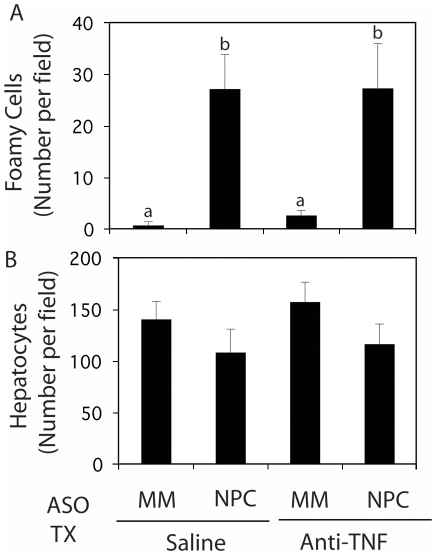
Effect of NPC1 knockdown and anti-TNF treatment on the number of foamy macrophages and hepatocytes. Quantification of foamy macrophages (A) and hepatocytes (B) in H&E stained liver sections from mice injected for 9 weeks with mismatched (MM) and NPC1-specific ASOs and subjected to treatment (TX) with saline or anti-TNF. Each bar represents the mean ± SD number of cells per field from 3 animals in each treatment group. The lettering (a, b) shows statistically dissimilar groups.

Sections of mismatched, control ASO-treated livers showed few foamy, vacuolated macrophages (seen in Fig. 2 and quantified in Fig. 3A). Saline and anti-TNF-treated liver sections were indistinguishable. NPC1 knockdown led to a 10-fold increase in the number of foamy and vacuolated macrophages. Knockdown of NPC1 in anti-TNF-treated mice led to the formation of the identical number of foamy macrophages. In both cases, the number of hepatocytes per field declined commensurate with the appearance of foamy macrophages (Fig. 3B).

Thus far there was only one indication that anti-TNF has a therapeutic effect on the NPC knockdown mice. That is that the liver weight did not increase significantly when NPC was knocked down in anti-TNF-treated mice. Next we examined the downstream consequences of hepatic lipid accumulation.

### Anti-TNF has a slight effect on hepatocyte apoptosis and stellate cell activation

**Figure 4:**
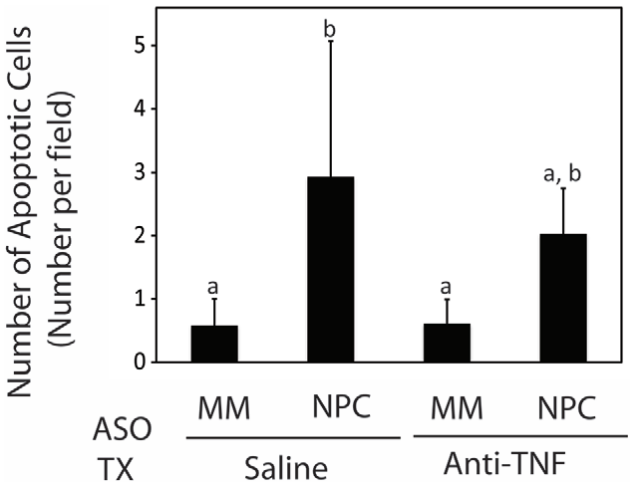
Effect of NPC1 knockdown and anti-TNF treatment on hepatic apoptosis. Liver sections from mice injected for 9 weeks with mismatched (MM) and NPC1-specific ASOs and subjected to treatment (TX) with saline or anti-TNF were subjected to fluorometric TUNEL staining. Apoptotic nuclei were quantified. Each bar represents the mean ± SD number of cells per field from 5 animals in each treatment group. The lettering (a, b) shows statistically dissimilar groups. Samples with two letters represent values that are intermediate to the statistical groups and are thus not considered significantly different from either group.

Knockdown of NPC1 was shown previously to result in a 10-fold increase in the number of apoptotic hepatocytes, which was offset by proliferation of hepatic stellate cells [11]. In this study, we found 0.6 apoptotic cells per field in TUNEL-stained liver sections from mice injected with mismatched ASOs and treated with either saline or anti-TNF (Fig. 4). The number of apoptotic cells increased 5-fold by NPC1 knockdown (p<0.05). Anti-TNF treatment provided some protective effect in that NPC1 knockdown in anti-TNF-treated mice led to an intermediate 3-fold increase in the number of apoptotic cells.

**Figure 5:**
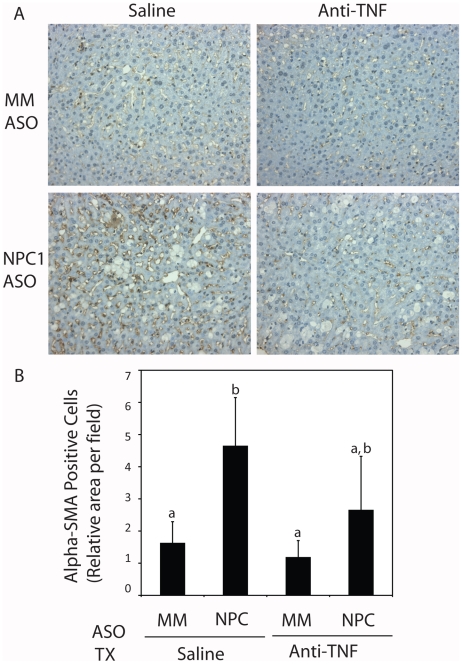
Stellate cell activation after NPC1 knockdown and anti-TNF treatment. Liver sections from mice injected for 9 weeks with mismatched (MM) and NPC1-specific ASOs and subjected to treatment with saline or anti-TNF were subjected to α-smooth muscle actin immunohistochemistry (A). Immunohistochemical reaction product was quantified (B). Each bar represents the mean± SD relative area of brown reaction product per field from 5 animals in each treatment group. The lettering (a, b) shows statistically dissimilar groups. Samples with two letters represent values that are intermediate to the statistical groups and are thus not considered significantly different from either group.

NPC1 knockdown also results in increased proliferation of hepatic stellate cells, which can be visualized by α-smooth muscle actin immunohistochemistry (Fig. 5A). Quantification of the α-smooth muscle actin-positive cells revealed a 3-fold increase in stellate cells when NPC1 was knocked down in saline-treated mice (p<0.05). Again, anti-TNF treatment of NPC1 knockdown mice led to a smaller increase in proliferating stellate cells (Fig. 5B).

Thus, the second and third indications that anti-TNF had a therapeutic effect were that the numbers of apoptotic cells and proliferating stellate cells did not increase significantly when NPC1 was knocked down in anti-TNF treated mice.

## Discussion

The goal of the current study was to evaluate an anti-TNF-α monoclonal antibody as a potential therapeutic modality for NPC disease. Our previous results had shown that TNF-α mediates some of the secondary consequences of NPC disease in the liver. NPC1 knockdown mice showed hepatomegaly, liver cholesterol accumulation, elevated serum liver enzymes, increased hepatocyte apoptosis and activation of stellate cells [11]. NPC1 knockdown in mice lacking TNF-α showed less clustering of macrophages into lipogranulomas, fewer apoptotic cells, reduced fibrosis and fewer activated stellate cells [12]. Thus, the lack of TNF appeared to slow the progression of NPC liver disease. Our hypothesis was that anti-TNF treatment of NPC1 knockdown mice would lead to a similar moderated phenotype.

Our results show that, as expected, anti-TNF had no effect on the primary NPC phenotype. NPC1 knockdown mice treated with vehicle or anti-TNF showed the same hepatic cholesterol storage, number of foamy cells throughout the hepatic parenchyma and release of liver enzymes into the blood. Anti-TNF treatment of NPC1 knockdown mice led to fewer apoptotic hepatocytes and activated stellate cells than did vehicle treatment; however, the attenuation did not reach statistical significance. Instead, anti-TNF treatment led to a phenotype that was intermediate between control and NPC1 knockdown.

NPC is a lysosomal storage disease with a primary impairment in intracellular cholesterol trafficking [15]. The defective export of cholesterol from lysosomes leads to a cellular accumulation of lipid-laden storage bodies and a deficiency of cholesterol transfer to the plasma membrane. NPC patients exhibit progressive neurodegeneration and hepatosplenomegaly, and have limited therapeutic options [16]. Several lines of evidence indicate that inflammation plays a role in the secondary consequences of the disease. The NPC brain and liver show increased expression of TNF-α and other members of the pro-inflammatory TNF-α pathway [6], [17], [18], [19].

Here we tested whether anti-TNF treatment would be efficacious for NPC patients. The monoclonal antibody that we used in our study binds and neutralizes murine TNF-α; it was a surrogate for infliximab, a monoclonal antibody that binds the membrane-bound and soluble forms of human TNF-α and prevents their binding to cellular receptors [20]. Infliximab (Remicade®) has been approved by use in patients with immune-mediate inflammatory diseases, such as Crohn's disease, rheumatoid arthritis, and psoriasis. In NPC1 knockdown mice, anti-TNF reduced some of the downstream effects of inflammation in the liver, but was not as effective as genetic knockout of TNF-α expression [12]. The weak effect of anti-TNF was seen when treatment was administered coincident with NC1 knockdown and prior to the onset of NPC liver disease. We expect it would have less benefit when given to NPC patients, who are typically diagnosed after tissue damage has taken place. These results may presage the need for small molecular inhibitors of the TNF-α pathway that are capable of entering tissues and crossing the blood-brain barrier.

## Materials and Methods

### Antisense Oligonucleotides

The 20-mer 2′-0-methoxyethyl modified ASOs were synthesized and purified as described previously [21], [22]. The sequence of the ASO targeted to the NPC1 mRNA is: 5′CCCGATTGAGCTCATCTTCG3′. As a control, we used an ASO with a mismatched sequence 5′CCTTCCCTGAAGGTTCCTCC3′. ASOs were dissolved in 0.9% saline and stored at −20°C.

### Animal Care and Treatment

Ethics Statement: All of the following procedures were approved by the Institutional Animal Care and Use Committee at Tufts University (Animal Welfare Assurance Number A-3775-01, protocol #71-06) and were in compliance with the NIH *Guide for the Care and Use of Laboratory Animals*.

Female C57BL/6 mice (4 wks of age) were purchased from The Jackson Laboratory (Bar Harbor, ME). They were housed 5 animals per cage and fed rodent chow. Mice were challenged with LPS by intraperitoneal injection of 200 µl of a 1-mg/ml solution (Sigma L5293). Mice were injected intraperitoneally with either NPC1 ASO or a mismatched control ASO at a dose of 100 mg/kg/wk for 9 weeks. They were also injected intraperitoneally with either saline or an anti-TNF monoclonal antibody (CNTO5048) at a dose of 12 mg/kg/wk for 9 weeks. At the end of the treatment period, animals were fasted overnight, then euthanized and blood samples taken by cardiac puncture. Mice were perfused with cold PBS via cardiac puncture, after which tissues were dissected and fixed in 10% formalin or snap-frozen in liquid nitrogen.

### Immunoblots

Liver tissue was blotted for NPC1 as previously described [11] using an NPC1 antibody from Abcam, Cambridge, MA. Anti-β-actin monoclonal antibody (Sigma) was used at 1∶15,000 dilution.

### Measurement of Cholesterol Content

Aliquots of 400 µg liver homogenate were subjected to Folch extraction [23]. Stigmasterol (45 µg) was added to each sample as an internal standard. The Folch organic phase was injected into a Hewlett-Packard 5890 gas chromatograph with a DB-17 capillary column (15 m×0.53 mm, Alltech) held at 255°C.

### Serum Chemistries

Blood was allowed to clot for 30 min, then subjected to centrifugation at 1000× *g* for 30 minutes. Serum was sent to IDEXX Laboratories (Grafton, MA) for analysis of liver enzymes.

### Histology

Liver tissue was fixed in 10% formalin and paraffin embedded. Four-micron sections were cut and stained with Masson Trichrome, hematoxylin-eosin or anti-α smooth muscle actin by the Tufts Animal Histology Core or the Tufts Medical Center Histology Department. Slides were viewed using a Zeiss Axioplan microscope with a 20X objective. The number of hepatocytes and foamy macrophages were quantified per field. α-Smooth muscle actin immunohistochemistry was quantified using ImageJ. For all analyses, 10 random fields were examined per liver; 3–5 animals were evaluated per treatment group.

### Detection of Apoptotic Cells

Apoptotic cells in formalin-fixed, paraffin-embedded liver tissue sections were detected using the DeadEnd Fluorometric TUNEL System (Promega, Madison, WI) according to the manufacturer's protocol. The number of apoptotic cells was quantified per field. Ten random fields were examined per liver; 5 animals were evaluated per treatment group.

### Statistical Analysis

All values are expressed as means ± SD. Statistical analysis was performed by one way ANOVA followed by Tukey's honestly significant differences test using the Vassar Stats website.
